# Integrating the Thrifty Genotype and Evolutionary Mismatch Hypotheses to understand variation in cardiometabolic disease risk

**DOI:** 10.1093/emph/eoae014

**Published:** 2024-07-31

**Authors:** Layla Brassington, Audrey M Arner, Marina M Watowich, Jane Damstedt, Kee Seong Ng, Yvonne A L Lim, Vivek V Venkataraman, Ian J Wallace, Thomas S Kraft, Amanda J Lea

**Affiliations:** Department of Biological Sciences, Vanderbilt University, Nashville, TN, USA; Department of Biological Sciences, Vanderbilt University, Nashville, TN, USA; Department of Biological Sciences, Vanderbilt University, Nashville, TN, USA; Department of Anthropology, University of Utah, Salt Lake City, Utah, USA; Department of Medicine, Faculty of Medicine, Universiti Malaya, Kuala Lumpur, Malaysia; Department of Parasitology, Faculty of Medicine, Universiti Malaya, Kuala Lumpur, Malaysia; Department of Anthropology and Archaeology, University of Calgary, Calgary, Alberta, Canada; Department of Anthropology, University of New Mexico, Albuquerque, New Mexico, USA; Department of Anthropology, University of Utah, Salt Lake City, Utah, USA; Department of Biological Sciences, Vanderbilt University, Nashville, TN, USA

**Keywords:** Thrifty Genotype Hypothesis, Evolutionary Mismatch Hypothesis, genotype x environment interactions, Orang Asli

## Abstract

More than 60 years ago, James Neel proposed the Thrifty Genotype Hypothesis to explain the widespread prevalence of type 2 diabetes in Western, industrial contexts. This hypothesis posits that variants linked to conservative energy usage and increased fat deposition would have been favored throughout human evolution due to the advantages they could provide during periods of resource limitation. However, in industrial environments, these variants instead produce an increased risk of obesity, metabolic syndrome, type 2 diabetes, and related health issues. This hypothesis has been popular and impactful, with thousands of citations, many ongoing debates, and several spin-off theories in biomedicine, evolutionary biology, and anthropology. However, despite great attention, the applicability and utility of the Thrifty Genotype Hypothesis (TGH) to modern human health remains, in our opinion, unresolved. To move research in this area forward, we first discuss the original formulation of the TGH and its critiques. Second, we trace the TGH to updated hypotheses that are currently at the forefront of the evolutionary medicine literature—namely, the Evolutionary Mismatch Hypothesis. Third, we lay out empirical predictions for updated hypotheses and evaluate them against the current literature. Finally, we discuss study designs that could be fruitful for filling current knowledge gaps; here, we focus on partnerships with subsistence-level groups undergoing lifestyle transitions, and we present data from an ongoing study with the Orang Asli of Malaysia to illustrate this point. Overall, we hope this synthesis will guide new empirical research aimed at understanding how the human evolutionary past interacts with our modern environments to influence cardiometabolic health.

## INTRODUCTION

Some of the largest public health burdens today can be collectively characterized as cardiometabolic diseases, encompassing cardiovascular diseases (e.g. ischemic heart disease, stroke), type 2 diabetes, and their major risk factors (e.g. obesity, hypertension). Rates of cardiometabolic diseases have remained high in high-income countries like the USA for several decades, and while prevalence has historically been low in low- and middle-income countries, it has skyrocketed in recent years [1]. Population-specific risk and rapid increases in only a few generations imply that environmental factors play a major role in pathophysiology. On the other hand, cardiometabolic diseases also have a clear genetic basis: for example, many contributing loci have now been mapped for type 2 diabetes [2, 3], including those that interact with environmental factors in the form of genotype × environment (GxE) interactions [4].

While it may seem obvious and unsurprising today that environmental, genetic, and interactive processes create variation in complex disease risk, this was less clear in 1962 when James Neel introduced the now famous Thrifty Genotype Hypothesis (TGH) [5]. The TGH was aimed at providing an explanation for why type 2 diabetes—which was known to run in families at the time and thus assumed to have a genetic basis—was at relatively high frequency in industrial compared to non-industrial contexts [6]. To answer this question, Neel proposed an evolutionary explanation based on GxE interactions: he posited that genotypes predisposing individuals to high blood insulin levels would have been beneficial in energy-limited pre-industrial contexts. Specifically, this thrifty genotype would have had a selective advantage by favoring fat storage when food was abundant and thus improving survival in subsequent periods of scarcity. However, in post-industrial environments, where food is often abundant, individuals with the thrifty genotype are instead predisposed to type 2 diabetes and other cardiometabolic health issues.

**Figure 1.**
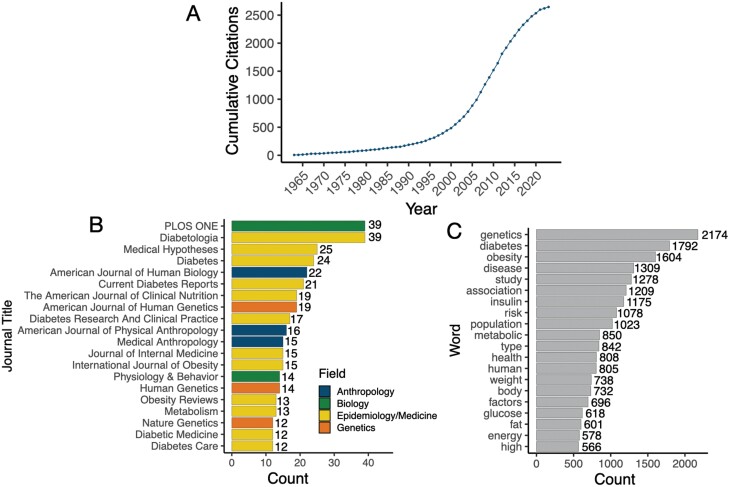
**Impact of James Neel’s Thrifty Genotype Hypothesis on the literature. (A)** The number of cumulative citations over time for the TGH 1962 paper. **(B)** Number of citations for the TGH 1962 paper per journal and colored by journal field (the top 20 journals are shown for ease of visualization). **(C)** Most common words from the abstracts of papers that have cited the TGH 1962 paper (the top 20 words are shown for ease of visualization).

The TGH was one of the first and most compelling attempts to provide an evolutionary explanation for genetic and environmental patterns of cardiometabolic disease. It quickly caught on and has since been widely cited, discussed and critiqued across diverse disciplines (**Fig. 1**; Supplementary Table 4). For example, as of December 2023, Neel’s hypothesis [5] has received 2646 citations, translating to an average rate of 40 citations per year (Fig. 1A). Only 20% of citations occurred in the first 40 years of the paper’s lifespan, with the remaining 80% occurring since 2002, emphasizing the endurance and increasing relevance of his ideas (Fig. 1). Further, the TGH has been widely cited in journals relevant to medicine (60% of publications) but has also had impacts on the fields of biology, anthropology, and genetics (Fig. 1). The hypothesis has also inspired a large number of related ideas, as well as revisions and expansions by Neel himself. In this review, we (i) trace the original formulation of the TGH and (ii) explore hypotheses sparked by the TGH that have persisted within biomedicine, evolutionary biology, and anthropology. In particular, we argue that the now popular Evolutionary Mismatch Hypothesis, which also emphasizes GxE interactions between previously beneficial alleles and post-industrial lifestyles, is in many ways a reimagining and expansion of the TGH. We also (iii) discuss empirical evidence that either exists or is needed; in service, we focus on how partnerships [7] with populations living non-industrial lifestyles could be particularly beneficial for filling current knowledge gaps, and we present data from our ongoing study with the Orang Asli of Malaysia to illustrate this point.

## THE THRIFTY GENOTYPE HYPOTHESIS: ORIGINAL FORMULATION, FOLLOW-UPs AND CRITIQUES

In the original formulation of the TGH, Neel proposed that famines were common enough throughout human pre-industrial history for natural selection to act on and favor thrifty genotypes linked to phenotypes that were ‘exceptionally efficient in the intake and/or utilization of food’ [6]. These genotypes would have conferred survival and/or fecundity advantages during times of scarcity by increasing the likelihood of energy availability, especially in the form of stored energy (i.e. fat). Neel further proposed that if thrifty genotypes were selected for high frequency in pre-industrial environments, this could explain the high prevalence of cardiometabolic disorders in populations that had recently transitioned to industrial contexts characterized by easier access to calories.

This overall idea has been very impactful, but theoretical, empirical, and ethical critiques about specific aspects of the TGH have since followed (see also a discussion of alternative hypotheses in Box 1). It is worth noting that several of the main critiques of the TGH were acknowledged and addressed by Neel himself in follow-up articles published decades later [8, 9]. First, there are several critiques that were largely products of what was known at the time the TGH was proposed. For example, Neel did not distinguish between type 1 and type 2 diabetes, which were not clinically well defined in the 1960s. Neel later clarified that his hypothesis was relevant to what is now known as type 2 diabetes, as well as obesity, hypertension and metabolic syndrome [9]. Additionally, the original hypothesis assumed a very simple genetic architecture for type 2 diabetes (e.g. a single locus of major effect) and generally oversimplified the complex interactions between genes, environment and lifestyle. These errors were acknowledged by Neel as an unavoidable byproduct of 1960s genetic knowledge, as our understanding of polygenicity and GxE interactions for complex traits were generally limited in the pre-genomic era [9].

Box 1 **Alternative and related evolutionary hypotheses**

There have been a few alternative and competing hypotheses in response to the TGH. These hypotheses focus on explaining high rates of cardiometabolic disease in post-industrial contexts through other mechanisms, and include the Drifty Genotype, Predation Release, and Thrifty Phenotype Hypotheses. We note that this list of related hypotheses is not exhaustive, nor do all hypotheses have similar empirical support. In particular, the Drifty Genotype and Predation Release Hypotheses are not empirically investigated or well supported at this time, while the Thrifty Phenotype Hypothesis has been a centerpiece of much evolutionary and biomedical work.

The Drifty Genotype Hypothesis, introduced by Speakman in 2008 [18], posits that genetic variants that increase susceptibility to metabolic disorders arise randomly and persist in populations due to genetic drift, rather than positive selection. This hypothesis suggests that these genetic variants are not necessarily adaptive or beneficial, but rather represent neutral or slightly deleterious mutations that become more common due to chance events in the absence of selection pressures [19, 82].

The Predation Release Hypothesis, introduced by Speakman in 2007 [83], proposes that there was not sufficient selection pressure for thrifty genotypes to persist during human evolutionary history. Instead, early humans may have experienced stabilizing selection against obesity due to the risk of predation. The loss of predation has therefore led to genetic drift and the rise of obesity rather than directed selection [83].

The Thrifty Phenotype Hypothesis, introduced by Hales and Barker in 1992 [84], suggests that metabolic and structural adaptations in response to early life undernutrition are often beneficial for early survival but might increase the risk of chronic diseases, such as type 2 diabetes, later in life [85, 86]. The Thrifty Phenotype Hypothesis has been widely researched and discussed, with general empirical support. For example, a review of the Thrifty Phenotype Hypothesis found evidence for ~30 different populations where, at least cross sectionally, there is a relationship between low birth weight (a proxy for fetal malnutrition) and increased incidence of metabolic syndromes later in life [85].

There were also potential theoretical issues in the original argument, which relied on the premise that pre-industrial populations regularly experienced famines or food shortages. Recent empirical tests of this idea using cross-cultural ethnographic data from hunter-gatherers have failed to find supporting evidence [10, 11]. Rather than being inherently prone to food insecurity, hunter-gatherers are well-suited to avoiding famine by adopting flexible, diverse and mobile foraging strategies. Hunter-gatherers also share food widely under most conditions, operating with what amounts to pooled energy budgets. Under starvation conditions, this cooperative strategy would have the effect of reducing inter-individual variation in mortality. Others have proposed that selection on thrifty genes was more likely after the appearance of agriculture. For example, Speakman [12] provides a critical review of these ideas, noting that the current state of evidence paints a far more complex picture of links between famine, mortality and body fat reserves than the TGH would suggest.

Another potential theoretical gap is that the TGH focused on the role of famine as a selective force, rather than broader energetic constraints specific to the human lineage. In particular, relative to other great apes, humans have very energetically costly bodies due to our large brains, somatic repair/maintenance requirements of long lifespans, high physical activity levels and high reproductive rates in natural fertility contexts [12–15]. These traits evolved in environments where food was limited (though not necessarily scarce or tied to frequent famine), and thus, it has been argued that our species as a whole may have evolved some level of thriftiness. For example, humans have evolved traits to buffer against and counter energy limitation [13], including a propensity to maintain higher body fat stores than other primates [14, 15], potentially a greater ability to accumulate body fat [16], and neurological mechanisms promoting fat- and sugar-seeking behavior [17]. Thus, thanks to our shared evolutionary history, effectively, all humans are vulnerable to chronic positive energy balance and cardiometabolic disorders in modern industrial and post-industrial environments in which energy from food is dependable and abundant.

Finally, there are ongoing discussions, primarily in the field of anthropology, about the ethical legacy of the TGH and the ways in which it has been interpreted (or misinterpreted) since its introduction. Although the language used in Neel’s original writings to describe non-industrial and recently industrialized populations was commonplace at the time, much of it would now be considered racialized and essentializing [18, 19, 20]. For example, in 1962 Neel calls for physiological and genetic studies of ‘primitive’ (a word with connotations of value judgment and inferiority [21]) hunter-gatherers to compare to data from industrialized societies like the USA. This rather binary comparison framework has been viewed by some as overly simplistic [11, 22–24], with the focus on genetic explanations for disease in Indigenous groups as encouraging ideas of genetic determinism [23, 25]. Further, in the original paper, lifestyle change among non-industrial societies is characterized as neutral to potentially positive (e.g. ‘the blessings of civilization’) without acknowledgement of fundamental causes and consequences such as colonization, forced assimilation, racism, poverty, unequal access to health care and education and environmental exploitation. The TGH is one (of numerous) examples from the fields of biomedicine, genetics and anthropology about how normative language and frameworks can change over time, but we bring up this set of critiques because we believe it is important to have critical discussions of our histories in order to acknowledge and work to overcome them today.

## EXPANDING THE THRIFTY GENOTYPE HYPOTHESIS INTO THE EVOLUTIONARY MISMATCH HYPOTHESIS

While valid issues and critiques have been raised about the TGH, the core of the original idea—that genetic variation selected for in previous environments generates cardiometabolic disease when placed against the backdrop of industrial diets and lifestyles—remains compelling. This concept has been expanded in the evolutionary medicine literature into what is now known as the Evolutionary Mismatch Hypothesis, which was first popularized in 1994 by Nesse and Williams [26]). The Evolutionary Mismatch Hypothesis posits that traits that evolved in past environments may no longer be advantageous if the environment rapidly shifts and individuals find themselves in a novel (e.g. industrial or post-industrial) environment to which their traits are mismatched [27, 28]. In essence, the Evolutionary Mismatch Hypothesis—which has been widely invoked to explain the high prevalence of various chronic non-infesctious diseases and other disorders in high-income countries [27]—is an expanded version of the TGH that is not necessarily specific to type 2 diabetes or cardiometabolic diseases. In our opinion, this connection to the concept of evolutionary mismatch, a core tenet of the field of evolutionary medicine, is perhaps the most enduring legacy of the TGH.

Neel and later others proposed several predictions about cardiometabolic disorders that follow from the TGH and mismatch more broadly. First, mismatch explanations for cardiometabolic disorders require a genetic basis. In other words, some (or all) individuals must carry a genotype, or more realistically, a polygenic genetic profile, that predisposes them to cardiometabolic disease in modern contexts. This prediction is clearly supported. For example, genome-wide association studies (GWAS) have identified hundreds of loci associated with visceral adipose tissue, type 2 diabetes, hypertension and cardiovascular disease [29–31]. Further, genome-wide genetic profiles, operationalized as polygenic risk scores, can (albeit weakly) predict type 2 diabetes, obesity and related cardiometabolic disorders [32].

Second, if interactions between previously selected genetic variants and modern lifeways generate cardiometabolic disorders, we would expect differences in the frequency of these conditions both within and between non- versus post-industrial populations. This prediction is also supported by a large and increasing body of research showing minimal obesity, hypertension, type 2 diabetes, metabolic syndrome and cardiovascular disease across modern-day hunter-gatherer, horticulturalist and other subsistence-level groups [33–36]. For example, Tsimane forager-horticulturalists have the lowest levels of coronary artery disease observed in any study to date (all comparative datasets are from post-industrial contexts) [34], while Hadza hunter-gatherers exhibit low levels of obesity and hypertension even into old age [33, 37]. Further, this body of research has shown that the low levels of cardiometabolic disease in subsistence-level settings can be largely attributed to lifestyle features that more closely recapitulate the environment in which humans evolved, such as diets that minimally contain processed foods and high levels of physical activity [38–40].

Third, if previously selected variants are now disease-causing, risk alleles in modern contexts should show signatures of past selection. In support, a now well-known study of Samoan Islanders identified a missense variant in CREBRF, which shows signatures of past selection, exhibits a positive association with body mass index and obesity in modern-day populations, and in functional experiments appears to alter lipid biology and accumulation [41]. A follow-up study performed in Polynesians associated the same missense variant in CREBRF with early insulin release, further implicating the gene in metabolic phenotypes [42]. In addition to this single locus example, there is also some evidence for generalizable patterns. For example, Byars *et al*. examined a genomic dataset of worldwide populations (HapMap) and found that positive selection consistently targeted present-day coronary artery disease variants more often than expected by chance [43]. In a similar spirit, Southam *et al*. examined all known type 2 diabetes and obesity-associated variants at the time (*n* = 30 loci in 2009), but did not find a similar pattern [44]; this is surprising in light of the findings of Byars *et al*., case studies like CREBRF, as well as other work on human complex traits more generally [45, 46]. More empirical work related to this prediction is needed.

Finally, the most compelling evidence for mismatch would be if disease-causing variants in post-industrial environments were also shown to be beneficial in non-industrial contexts that better reflect human evolutionary history. In other words, we would expect to see evidence for GxE interactions when comparing genetic effects on cardiometabolic traits in subsistence-level/energy-constrained environments versus post-industrial/energy-abundant environments. While there is clear evidence that GxE interactions exist and regulate cardiometabolic and other complex traits, the specific evidence we describe has not been systematically gathered thus far. Candidate gene studies offer some hints: for example, the apolipoprotein E4 allele (APOE4) is associated with increased cardiovascular and neurological disease risk in post-industrial populations, but in Tsimane forgers—who inhabit an energy limited and high pathogen environment—this same allele confers potential benefits by reducing innate inflammation with no downstream increase to cardiovascular disease risk [47]. In our opinion, the systematic lack of genome-wide studies of GxE interactions in non-industrial contexts is a symptom of the lack of large-scale genomic studies of all kinds with these types of populations. Filling this gap is essential for addressing inequality in biomedical studies and for moving knowledge forward, as discussed in the next sections.

## FILLING CURRENT KNOWLEDGE GAPS

As stated above, the evidence for the first two predictions—that cardiometabolic disorders have a genetic basis, and that non- versus post-industrial populations differ in the frequency of these conditions—is relatively robust. The few knowledge gaps that do exist are (i) studies of the genetic basis of cardiometabolic disorders are highly biased toward post-industrial contexts, and (ii) a handful of well-studied subsistence-level societies make up the bulk of our knowledge about health in non-industrial contexts (e.g. Tsimane forager-horticulturalists in Bolivia [48], Hadza hunter-gatherers in Tanzania [33]). While we do not expect expanded research in these areas to radically change any of the overall conclusions discussed above, filling these gaps is nonetheless important for advancing a robust understanding of biological phenomena as well as global health equity.

The second two predictions are in need of more empirical attention. Additional studies focused on overlaps between past selection and modern-day disease alleles are needed, with a few key improvements to keep in mind. First, we should make sure that comparisons between the genetic basis of a given disease and the selection history of the associated variants are assessed in the same or related populations. Studies of this kind have historically merged summary statistics from large GWAS conducted on individuals of European ancestry (e.g. UK Biobank [49]) with positive selection statistics estimated from large global datasets (e.g. HapMap, 1000 Genomes) [43, 50, 51]. As a result, selection statistics may not be relevant to the population used to identify the risk allele and more population-specific analyses are warranted in this space. A second potential improvement is that current studies place a strong emphasis on analyzing evidence for past positive selection, but additional forces such as purifying, balancing, or stabilizing selection are also worthy of consideration [45, 52]. Third, it is important to keep in mind that typical GWAS and evolutionary methods cannot easily localize the specific allele associated with disease or past selection; thus, overlaps are commonly conducted at the locus or region level (e.g. [53, 54]). One alternative strategy is to instead move beyond select loci and regions to polygenic approaches that aggregate genome-wide evidence for selection (e.g. [53–55]); while these approaches obscure interesting deep-dives into individual genes with important evolutionary histories and biological functions (e.g. [41]) they are perhaps more faithful to the polygenic nature of complex traits [56].

As discussed above, in our opinion, the most compelling and currently lacking evidence for mismatch would be GxE interactions that manifest between subsistence-level/energy-constrained environments versus post-industrial/energy-abundant environments. While a handful of studies have attempted such comparisons, this literature thus far has focused on candidate genes [47], limited lifestyle gradients within high-income countries [57, 58], and/or comparisons that confound genetic background with the environmental contrast of interest [59]. We believe there are a few alternative and fruitful paths forward. First, tractable model systems could be leveraged. For example, work in experimental evolution has already evolved organisms such as bacteria and fruit flies to resource limitation as well as feast-famine cycles explicitly [60, 61]. These constraint-adapted populations could be exposed to ‘novel’ conditions such as a high sugar diet or resource abundance in a design that would be well-suited to map GxE interactions and test mismatch principles [62]. Specifically, in such systems we would be able to test whether variants involved in adaptation to resource constraint exhibit GxE interactions in more resource-abundant environments in ways that impact metabolic physiology. Similarly, the culture of isolated human cells in different in vitro environments, followed by evaluation of molecular phenotypes and GxE mapping, could be leveraged for similar questions. This molecular QTL design has been well-exploited for understanding how genetic effects on immune cell traits change when cells are or are not infected [63–66] but has not been explicitly leveraged in mismatch frameworks for cardiometabolic disease. Studies that examine genetic effects in adipocytes or other cardiometabolic cell types exposed to relevant stimuli, such as particular dietary components common in post-industrial contexts [67], are entirely within reach.

To move beyond model and in vitro systems and directly map GxE interactions in humans, we would need paired genomic and health data collected from genetically similar individuals exposed to a spectrum of subsistence-level/energy-constrained to post-industrial/energy-abundant environments. This is a tall order, but not infeasible. In particular, essentially all modern-day subsistence-level groups are facing increasing threats to their traditional lifeways as a result of colonization, globalization, urbanization and acculturation. Thus, populations undergoing rapid lifestyle transitions provide opportunities to observe the effects of previously selected variants in environments of energy-constraint versus plenty. Importantly, the health effects of this lifestyle change are already at the forefront of current research in biological anthropology, public health and related fields, as individuals exposed to market integration and acculturation are consistently experiencing worse cardiometabolic health relative to their more traditional-living counterparts [35, 68, 69]. We believe the time is now to integrate this work with genomic analyses to understand mismatch and GxE interactions, and more importantly to bolster efforts to improve health and well-being in these vulnerable groups [70]. Below, we describe our ongoing efforts with one group experiencing rapid lifestyle change—the Orang Asli of Malaysia—to explore a possible model.

## A CASE STUDY FROM THE ORANG ASLI HEALTH AND LIFEWAYS PROJECT

The Orang Asli are the Indigenous peoples of Peninsular Malaysia, comprising < 1% (~210,000 people) of Malaysia’s population. They are typically divided in 19 distinct ethnolinguistic groups and three broader sub-groups: the Semang (traditionally nomadic hunter-gatherers), the Senoi (traditionally horticulturalists) and the Proto-Malay (traditionally mixed subsistence practitioners). The Orang Asli show genomic evidence of a long history in their ancestral environments [71], and their ecology and biometric phenotypes have been well studied by anthropologists for several decades [72, 73].

Over the last 50 years, Orang Asli in Malaysia have undergone rapid lifestyle change due to government-promoted assimilation, deforestation as a result of natural resource extraction and the expansion of built infrastructure [74]. Due to these trends, individuals of similar genetic backgrounds now live in both relatively traditional and urban settings, and these changes have occurred within the last 1–2 generations. Urban transitions have been previously found to adversely impact Orang Asli cardiometabolic health [75], and cardiometabolic diseases are a major concern for Orang Asli communities.

**Figure 2.**
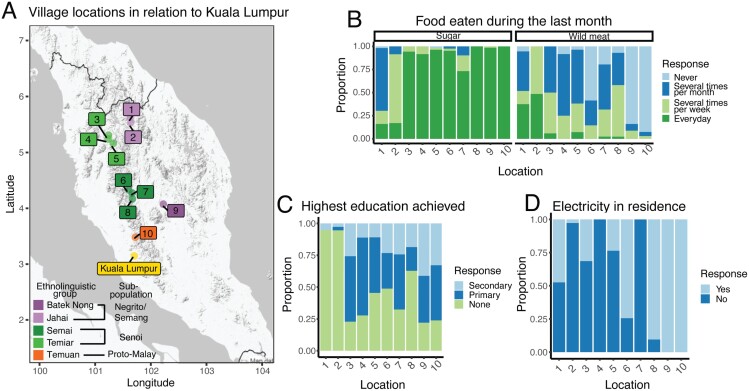
**Orang Asli communities span a lifestyle gradient. (A)** Ten Orang Asli villages previously visited by the Orang Asli Health and Lifeways Project. To date, the project has focused on five ethnolinguistic groups across all three subpopulations. For each location, the primary (> 70%) ethnolinguistic group is labeled, but we note that there is heterogeneity. The location of Kuala Lumpur, the capital of Malaysia, is also shown. Each of the following plots is ordered from highest to lowest latitude, indicative of their distance from Kuala Lumpur, with villages on the left of the x-axis being the furthest from Kuala Lumpur. (B) The proportion of people from each community reporting different responses regarding how often they ate sugar and wild meat eaten within the last month. Wild meat is a staple of the traditional diet, while sugar, which can only be obtained through trade, is indicative of market integration. **(C)** The proportion of people from each community who reported their highest level of education. More education is indicative of a higher degree of acculturation. **(D)** The proportion of people from each community reporting presence or absence of electricity in their residence. We note that Selaor and Kelab are communities on the extreme of the lifestyle gradient, practicing very traditional lifestyles. The other eight villages we have partnered with are more intermediate lifestyles between traditional and novel.

To understand how the Orang Asli’s evolutionary past and current lifestyle interact to affect health, we have established the Orang Asli Health and Lifeways Project (OA HeLP) (following the guidelines of [7] and further described in [76]). To date, we have worked with over 600 individuals across 10 villages to assess evolutionary mismatch by investigating lifestyle change and its relationship to health (Fig. 2A; Supplementary Table 5). Our data emphasize an extreme and multifaceted gradient: at one end of the gradient, individuals primarily rely on wage labor for income, live in permanent concrete structures surrounded by a built environment, and have consistent access to market foods, labor-saving devices and motorized transportation. On the other end of the gradient, some Orang Asli continue to live very traditional lifestyles in bamboo houses surrounded by intact tropical rainforest, with a heavy reliance on foraging or small-scale horticulture and little access to government services or modern technology. For example, the proportion of individuals within a village who recently ate wild meat from the forest (a key component of a subsistence-level diet) ranges from 3% to 94%; similarly, the proportion of individuals having at least some primary education (indicative of acculturation) ranges from 5% to 78% (Fig. 2B). These extreme gradients broadly track from north to south, coinciding with development, urban infrastructure and contact with the dominant Malay culture (Fig. 2A and C). Indeed, we found that distance from Kuala Lumpur (KL), the capital city of Malaysia, significantly predicts these aspects of the lifestyle gradient, such that increased distance from KL was associated with a smaller sugar intake and a greater consumption of wild meat, lower level of education achieved and fewer visits to KL (linear models controlling for age and gender, all *P* < 0.05; Supplementary Table 1). This observed lifestyle gradient is critical for a robust study of evolutionary mismatch, as individuals with similar genetic backgrounds can be found across environments that are more matched versus mismatched to their recent evolutionary history.

**Figure 3.**
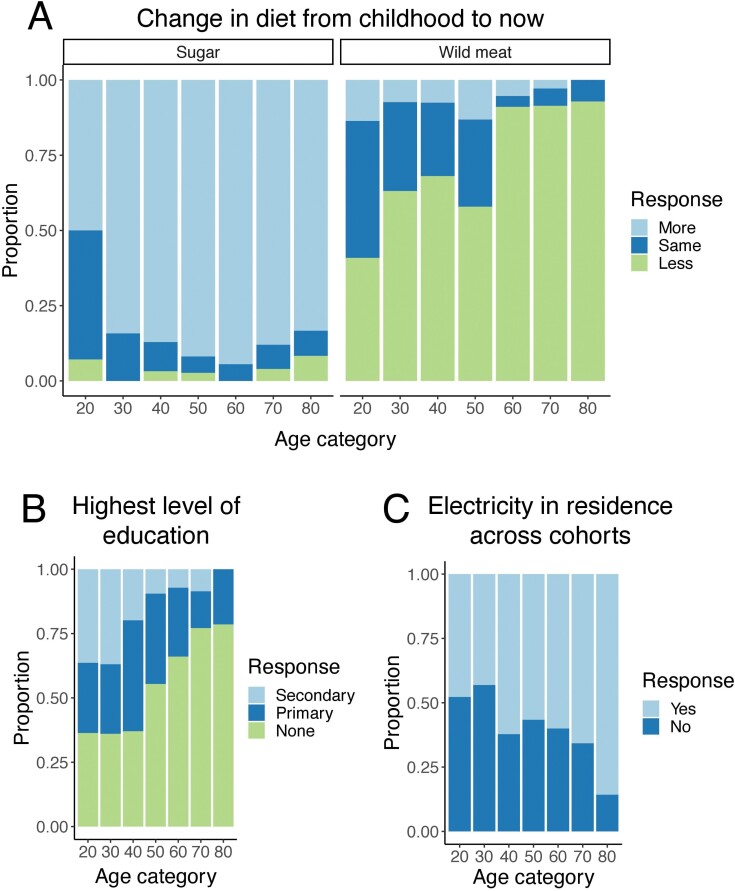
**Rapid generational lifestyle shift among the Orang Asli. (A)** Proportion of people in 10-year age bins who reported how their consumption of sugar and wild meat has changed from their childhood to now. Data show an overall increase in the consumption of sugar, which is obtained from markets, as well as a corresponding decrease in the consumption of wild meat across cohorts. **(B)** Proportion of people in ten year age bins who reported their highest level of education. The total proportion of individuals without any schooling increases across cohorts. **(C)** Proportion of people in 10-year age bins who reported the presence or absence of electricity in their residence.

Importantly, this lifestyle change has occurred within the last 1–2 generations, such that previously selected alleles have only recently been exposed to novel environments. For example, 93% of individuals aged 80–90 eat less wild meat now compared to when they were a child, emphasizing lifestyle change within a lifetime (Fig. 3A). Comparisons between age cohorts also demonstrate the rapid pace of lifestyle shifts: while only 21% of individuals aged 80-90 have had any schooling, 64% of individuals aged 20–30 have had at least primary level education (Fig. 3B). Our preliminary data also emphasize that indicators of cardiometabolic health vary widely across communities. For example, the mean waist circumference by village ranges from 68 to 93 cm in females and from 66 to 83 cm in males (Supplementary Fig. 1; Supplementary Table 2). Similarly, mean body fat percentage by village ranges from 18% to 34% in females and from 10% to 19% in males—an almost 2-fold difference between the highest and lowest values for both sexes (Supplementary Fig. 2; Supplementary Table 3). Using a linear model, we identified a significant negative relationship between distance from Kuala Lumpur and waist circumference as well as body fat percentage (Supplementary Table 1).

Together, the contemporary conditions faced by Orang Asli make it possible to test for TGH-inspired GxE interactions. Specifically, we are currently conducting whole-genome sequencing across this gradient in order to ask whether genetic variants that have been historically under positive selection and are linked to functional benefits in more traditional contexts instead put individuals at risk of cardiometabolic diseases in more acculturated, market-integrated contexts. While we highlight the lifestyle gradient among contemporary Orang Asli as one model for testing for TGH-inspired GxE interactions, we note that other research partnerships with subsistence-level communities undergoing lifestyle change are similarly poised. For example, a mismatch in sleep ecology and inactivity has been studied in the Hadza [77, 78], although these comparisons were made to industrialized populations with different genetic backgrounds, not Hadza living along a lifestyle gradient. However, comparisons among the Shuar, a mixed subsistence Amerindian population living in southeastern Ecuador, reveal associations between market integration, body size and cardiometabolic health. Here, increased market integration is associated with increased height, weight, high-density lipoprotein cholesterol and diastolic blood pressure when compared with individuals of the same genetic background with more traditional lifestyles [68, 79]. Studies among the Turkana, a pastoralist population living in northwest Kenya, and the Tsimane, a horticulturalist population living in Bolivia, take potential evolutionary mismatch research one step further by generating genetic data to identify regions in the genome under positive selection [54, 80]. In the future, integrative anthropological studies combined with genomic methods will be a powerful way to detect evolutionary mismatch occurring in contemporary populations.

## CONCLUSIONS AND FUTURE DIRECTIONS

Here, we have attempted to trace the influential TGH from its original formulation to its modern-day impact within evolutionary medicine and related fields. Like any hypothesis, the discourse around it has changed as perspectives on culture, biology and health have also changed, and it is useful to take stock of how interpretations and attitudes towards this hypothesis have transformed. The core of the TGH that our evolutionary past interacts with our present environments to impact cardiometabolic health, remains an exciting and important idea. Yet, despite the rich theoretical discourse that has taken place over the lifetime of this hypothesis, there is still much theoretical and empirical work to do to understand how these interactions play out to impact modern-day health. In particular, there remains a relative paucity of evidence for key TGH predictions, namely that cardiometabolic risk alleles are the product of past selection and that these alleles confer identifiable benefits in non-industrial environments.

Theoretically, we note that the hypothesis was (at least in part) motivated by a desire to understand the variable risk of metabolic dysregulation due to population-specific histories of natural selection and local adaptation. In our opinion, this is still a worthwhile goal, but the relevant selection pressures and specific predictions are in need of serious updating and expansion. In particular, Neel’s evolutionary arguments about population-specific risk relied on relatively simplistic narratives about recent famine in Indigenous lineages, rather than a principled framework about the quantifiable selection pressures that vary across all human populations and may select for different metabolic phenotypes. These likely include myriad factors such as ambient temperature/thermoregulation [81], environmental seasonality [13] and predictability [20], locomotor costs [71] and subsistence ecology (i.e. hunting and gathering vs. horticulture, availability of coastal resources, etc.). These are quantifiable features of human environments that could be used to build evolutionary models. In other words, although energy may have been limiting in many ancestral human environments, it is unlikely that the resulting constraints and tradeoffs would have been homogenous across the many environments colonized by humans. This history of localized selection pressures is important and likely relevant to population variation in metabolic traits today; however, new models need to move beyond cultural stereotypes to well-conceived theoretical models with testable predictions.

We hope the type of work we have proposed here will encourage others to test for GxE interactions in the context of TGH and mismatch. We believe that identifying GxE loci and understanding their biology is important for moving beyond false, stereotyped and overly simplistic links between particular populations or cultures and health. Instead, focusing on both selection and phenotypic effects at the genotypic level will allow us to understand mechanistic processes that are relevant to both evolution and biomedicine. We believe that partnering with small-scale subsistence-level populations can bring us one step closer to achieving this goal; in doing so, it will also help us build a globally representative understanding of genomic and biological phenomena, which are currently highly biased to individuals of European ancestry living in post-industrial contexts. We believe that individuals working within the evolutionary medicine community are especially well-poised to make these interdisciplinary connections in ways that will benefit both science and community health.

## Supplementary Material

eoae014_suppl_Supplementary_Tables_S1-S5_Figures_S1-S2

## Data Availability

OA HeLP’s highest priority is the minimization of risk to study participants. OA HeLP adheres to the ‘CARE Principles for Indigenous Data Governance’ (Collective Benefit, Authority to Control, Responsibility, and Ethics). OA HeLP is also committed to the ‘FAIR Guiding Principles for scientific data management and stewardship’ (Findable, Accessible, Interoperable, Reusable). To adhere to these principles while minimizing risks, individual-level data are stored in the OA HeLP protected data repository, and are available through restricted access. Requests for de-identified, individual-level data should take the form of an application that details the exact uses of the data and the research questions to be addressed, procedures that will be employed for data security and individual privacy, potential benefits to the study communities and procedures for assessing and minimizing stigmatizing interpretations of the research results. Requests for de-identified, individual-level data will require institutional IRB approval (even if exempt). OA HeLP is committed to open science and the project leadership is available to assist interested investigators in preparing data access requests (see orangaslihealth.org for further details and contact information). All scripts are available at https://github.com/audreyarner/thriftygenotype_review
