# Supplementary material for: Integrating the Thrifty Genotype and Evolutionary Mismatch Hypotheses to understand variation in cardiometabolic disease risk
Source: Evol Med Public Health. 2024 Jul 31;12(1):214–26. doi: 10.1093/emph/eoae014 (PMC11525211; doi:10.1093/emph/eoae014)
Supplement: eoae014_suppl_Supplementary_Tables_S1-S5_Figures_S1-S2 [file eoae014_suppl_supplementary_tables_s1-s5_figures_s1-s2.docx]

**Supplementary Materials for “Integrating the Thrifty Genotype and Evolutionary Mismatch Hypotheses to understand variation in cardiometabolic disease risk”**

Layla Brassington^1*^, Audrey M. Arner^1*^, Marina M. Watowich^1^, Jane Damstedt^6^, Ng Kee Seong^2^, Yvonne Ai Lian Lim^3^, Vivek V. Venkataraman^4^, Ian Wallace^5^, Thomas S. Kraft^6^, Amanda J. Lea^1,7^

*these authors contributed equally

^1^Department of Biological Sciences, Vanderbilt University, Nashville, TN, USA

^2^Department of Medicine, Faculty of Medicine, Universiti Malaya, Kuala Lumpur, Malaysia

^3^Department of Parasitology, Faculty of Medicine, Universiti Malaya, Kuala Lumpur, Malaysia

^4^Department of Anthropology and Archaeology, University of Calgary, Calgary, Alberta, Canada

^5^Department of Anthropology, University of New Mexico, Albuquerque, New Mexico, USA

^6^Department of Anthropology, University of Utah, Salt Lake City, Utah, USA

^7^Correspondance to amanda.j.lea@vanderbilt.edu

**Supplementary Methods**

Collection and analysis of citations

The collection of DOIs that have cited Neel’s 1962 Thrifty Genotype Hypothesis paper was aggregated using the citation chaser R Shiny app [[1]](https://paperpile.com/c/8gpguh/XfWQs). Here, we input the original paper and the automated output included the publication type, authors, publication title, publication year, abstract, publishing journal, publication volume and issue, and the DOI for the publication. We used this list of citations to calculate the cumulative number of citations over time. To determine the field for each journal, we went to the scope and aims page of each journal’s website to find keywords on how the journal describes themselves. We then used those keywords to bin each journal into one of four fields (anthropology, biology, epidemiology/medicine, and genetics; see SI Table 4). In order to count the most common words in each abstract, we used the R packages stringr and stopwords only including words in English and excluding the words "can", "may", "however", "found","also","whether", "suggest","show","na","=","2", and "de" [[2,3]](https://paperpile.com/c/8gpguh/0xmmW+vBvCn). Additionally, we combined the words “association” and “associated”, “disease” and “diseases”, “gene” and “genes” and “genetics”, “population” and “populations”, “studies” and “study” to eliminate redundancy.

Data from the Orang Asli Health and Lifeways Project

A detailed protocol for the Orang Asli Health and Lifeways Project is provided in [[4]](https://paperpile.com/c/8gpguh/77TTJ). The interview and health data summarized here were collected between June 2022 and October 2023 from self-reported Orang Asli individuals who were 18 years or older. During this time, researchers visited locations where Orang Asli individuals were known to reside. At each sampling location, the headman and community were first consulted about the project before individuals were invited to participate. After this, individual consent was obtained from each individual in their language of choice.

Structured interviews were conducted with all participants to collect information about both early life and current experiences, especially as these experiences relate to lifestyle, acculturation, market-integration, and urbanization. The following self-reported variables from the interviews are relevant to our analyses:

- Age
- Ethnolinguistic group
- Wild meat eaten in the last month
- Sugar eaten in the last month
- Amount of wild meat eaten now relative to in childhood (categorical variable: more/less/same)
- Amount of sugar eaten now relative to in childhood (categorical variable: more/less/same)
- Electricity in residence (categorical variable: yes/no)
- Ever visited Kuala Lumpur (capital city of Malaysia)
- Number of times visited Kuala Lumpur

For each analysis, individuals who did not answer the question of interest were removed. If individuals answered that they had never been to Kuala Lumpur, we set their number of visits to zero. We then binned individuals into those that have visited Kuala Lumpur once, between two and nine times, and ten times or more. To identify recent changes in lifestyle, we binned individuals into age groups of ten years starting from the age of 20. Due to sample size constraints, the oldest age group we included was individuals ages 80-89. The number of individuals in each age group ranged from 14 in age group 80-89 to 119 in age group 40-49 (SI Table 5). Body fat was collected using a digital bioelectrical impedance scale (TANITA's BC-558 FDA Cleared Ironman Segmental Body Composition Monitor). Waist circumference was measured with a tape measure. We used linear models controlling for age and sex to determine whether distance from KL (as measured by latitude) could predict each of four aspects of the lifestyle gradient: consumption of wild meat, consumption of sugar, highest education achieved, and number of visits to KL. We also used linear models controlling for age and sex to determine whether distance from KL (as measured by latitude) could predict health, specifically waist circumference and body fat percentage.

Procedures for this study have been reviewed and approved by the Medical Review and Ethics Committee of the Malaysian Ministry of Health (protocol ID: NMRR- 20- 2214- 55565), the Malaysian Department of Orang Asli Development (permit ID: JAKOA.PP.30.052 JLD 21 (98)) and the Institutional Review Boards of the University of New Mexico (protocol ID: 14420) and Vanderbilt University (protocol ID: 212175). Throughout the project, we have followed established principles for ethical biomedical research among Indigenous communities, including fostering collaboration, building cultural competency, being transparent about research practices, supporting capacity building, and disseminating research findings [[5]](https://paperpile.com/c/8gpguh/Akcsx).

**SI Figure 1: Variation in waist circumference by location.** Each point on the plot represents the mean waist circumference (in centimeters) at each location, with the bars representing the standard deviation. Locations are ordered from highest to lowest latitude, indicative of their distance from Kuala Lumpur, with villages on the left being furthest from Kuala Lumpur.

**SI Figure 2: Variation in body fat percentage by location.** Each point on the plot represents the mean body fat percentage at each location, with the bars representing the standard deviation. Locations are ordered from highest to lowest latitude, indicative of their distance from Kuala Lumpur, with villages on the left being furthest from Kuala Lumpur.

**Supplementary Table 1: Linear model outputs for distance to Kuala Lumpur (KL)**

| **Variable** | **Beta** | **P-value** |
| --- | --- | --- |
| Sugar consumption | -0.513 | <2 x10^-16^ |
| Wild meat consumption | 0.892 | <2 x10^-16^ |
| Highest education achieved | -0.536 | <2 x10^-16^ |
| Electricity in residence | -0.3848 | <2 x10^-16^ |
| Body fat percentage | -1.235 | 0.0324 |
| Waist circumference | -3.162 | 0.00118 |

**Supplementary Table 2: Orang Asli waist circumference by village**

| **Location** | **Sex** | **Mean** | **Standard Deviation** |
| --- | --- | --- | --- |
| 1 | Female | 79.99 | 10.18 |
|  | Male | 77.12 | 12.20 |
| 2 | Female | 73.62 | 9.04 |
|  | Male | 72.49 | 6.27 |
| 3 | Female | 80.16 | 12.69 |
|  | Male | 79.17 | 12.06 |
| 4 | Female | 76.33 | 4.13 |
|  | Male | 74.38 | 10.28 |
| 5 | Female | 84.65 | 10.30 |
|  | Male | 75.49 | 18.65 |
| 6 | Female | 81.48 | 13.33 |
|  | Male | 79.96 | 12.11 |
| 7 | Female | 68.52 | 12.81 |
|  | Male | 66.53 | 15.81 |
| 8 | Female | 82.13 | 10.41 |
|  | Male | 80.82 | 9.47 |
| 9 | Female | 84.54 | 15.47 |
|  | Male | 82.17 | 15.90 |
| 10 | Female | 93.38 | 13.26 |
|  | Male | 83.19 | 14.56 |

**Supplementary Table 3: Orang Asli body fat percentage by village**

| **Location** | **Sex** | **Mean** | **Standard Deviation** |
| --- | --- | --- | --- |
| 1 | Female | 29.39 | 8.70 |
|  | Male | 15.98 | 6.78 |
| 2 | Female | 19.16 | 5.52 |
|  | Male | 11.25 | 5.12 |
| 3 | Female | 30.47 | 8.54 |
|  | Male | 17.00 | 10.02 |
| 4 | Female | 23.04 | 5.65 |
|  | Male | 12.85 | 7.45 |
| 5 | Female | 32.44 | 6.14 |
|  | Male | 16.95 | 6.80 |
| 6 | Female | 27.57 | 8.43 |
|  | Male | 16.37 | 6.60 |
| 7 | Female | 18.35 | 7.53 |
|  | Male | 10.45 | 6.66 |
| 8 | Female | 27.20 | 6.80 |
|  | Male | 16.73 | 6.19 |
| 9 | Female | 30.94 | 8.98 |
|  | Male | 18.75 | 9.10 |
| 10 | Female | 34.51 | 7.85 |
|  | Male | 16.24 | 8.93 |

**Supplementary Table 4: Journal field designation**

**
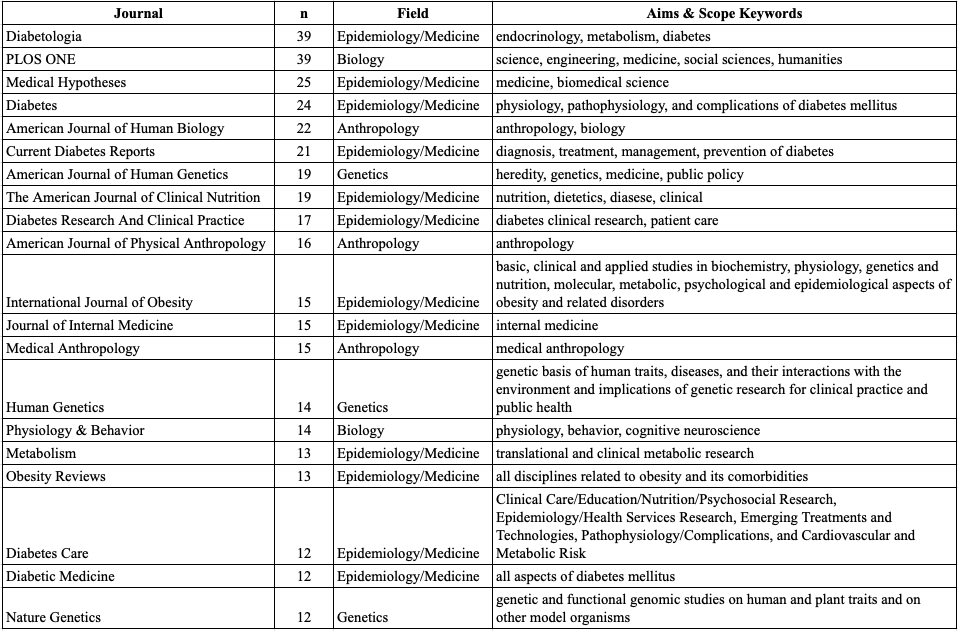
**

**Supplementary Table 5: Age group distributions**

| Age group | 20-29 | 30-39 | 40-49 | 50-59 | 60-69 | 70-79 | 80-89 |
| --- | --- | --- | --- | --- | --- | --- | --- |
| Number of individuals | 44 | 122 | 119 | 76 | 56 | 35 | 14 |

**Supplementary References**

1. [Creators Haddaway, N R Grainger, M. J. 1 Gray, C. T. 2 Show affiliations 1. @NINAnor 2. Newcastle University. citationchaser: An R package and Shiny app for forward and backward citations chasing in academic searching. doi:](http://paperpile.com/b/8gpguh/XfWQs)[10.5281/zenodo.4543513](http://dx.doi.org/10.5281/zenodo.4543513)

2. [Multilingual Stopword Lists [R package stopwords version 2.3]. 2021 [cited 11 Dec 2023]. Available:](http://paperpile.com/b/8gpguh/0xmmW) <https://cran.r-project.org/web/packages/stopwords/index.html>

3. [Wickham H, Others. Stringr: Simple, consistent wrappers for common string operations. 2019.](http://paperpile.com/b/8gpguh/vBvCn)

4. Garske KM, Pan DZ, Miao Z et al. Reverse gene-environment interaction approach to identify variants influencing body-mass index in humans. *Nat Metab* 2019.

5. Claw KG, Anderson MZ, Begay RL et al. A framework for enhancing ethical genomic research with Indigenous communities. *Nat Commun* 2018.
